# Spatial variability and depuration of tetrodotoxin in the bivalve *Paphies australis* from New Zealand

**DOI:** 10.1016/j.toxcx.2019.100008

**Published:** 2019-02-23

**Authors:** Laura Biessy, Kirsty F. Smith, D. Tim Harwood, Michael J. Boundy, Ian Hawes, Susanna A. Wood

**Affiliations:** a Cawthron Institute, Private Bag 2, Nelson, 7010, New Zealand; b Department of Biological Sciences, University of Waikato, Private Bag 3105, Hamilton, 3240, New Zealand; c New Zealand Food Safety Science & Research Centre, Palmerston North, 4442, New Zealand

**Keywords:** Biotoxin, Clam, Emerging threat, Geographic variability, Marine bivalves, Neurotoxin

## Abstract

Tetrodotoxin (TTX) is a potent neurotoxin responsible for many human intoxications globally. Despite its potency and widespread occurrence in taxonomically diverse species, the primary source of TTX remains uncertain. *Paphies australis*, an endemic clam found in New Zealand, has been found to contain TTX in several locations. However, it is unknown if this represents endogenous production or accumulation from an external source. To address this question, the concentrations of TTX in whole *P. australis* and dissected organs (siphons, foot, digestive gland and the ‘rest’) from thirteen sites around New Zealand were determined using liquid chromatography-tandem quadrupole mass spectrometry analysis (LC-MS/MS). Depuration rate of TTX was also investigated by harvesting and measuring concentrations in *P. australis* maintained in captivity on a toxin-free diet every three to 15 days for 150 days. The LC-MS/MS analyses of the spatial samples showed that TTX was present in *P. australis* from all regions tested, with significantly (*p* < 0.001) higher concentrations (15–50 μg kg^−1^) observed at lower latitudes of the North Island compared with trace levels (0.5–3 μg kg^−1^) in the South Island of New Zealand. Tetrodotoxin was detected in all the dissected organs but the siphons contained the highest concentrations of TTX at all sites analysed. A linear model of the depuration data identified a significant (*p* < 0.001) decline in total TTX concentrations in *P. australis* over the study period. The siphons maintained the highest amount of TTX across the entire depuration study. The digestive glands contained low concentrations at the start of the experiment, but this depurated rapidly and only traces remained after 21 days. These results provide evidence to suggest that *P. australis* does not produce TTX endogenously but obtains the neurotoxin from an exogenous source (e.g., diet) with the source more prevalent in warmer northern waters. The association of higher TTX concentrations in shellfish with warmer environments raises concerns that this toxin's distribution and abundance could become an increasing human health issue with global warming.

## Highlights

• TTX-containing *Paphies australis* were maintained in captivity for 150 days and significantly depurated the toxin.
• Thirteen populations of *Paphies australis* from around New Zealand were collected and tested for TTX.
• All populations tested contained TTX but a significant latitudinal gradient was observed.
• This study provides further evidence of an exogenous source of TTX in marine bivalves.

## 1 Introduction

Tetrodotoxin (TTX) is a potent neurotoxin that blocks voltage-gated sodium channels (Noguchi and Ebesu, 2001). Globally, it has been responsible for up to 100 human intoxications per annum (Isbister and Kiernan, 2005, Tsuda et al., 1964). It is named after the pufferfish family, Tetraodontiformes, from which it was first identified and isolated (Chau et al., 2011). Tetrodotoxin was initially thought to only occur in pufferfish but has now been identified in a wide range of marine, freshwater and terrestrial vertebrates and invertebrates (Bane et al., 2014). The source of TTX remains unknown and controversial: there are studies which provide evidence to support an exogenous source such as bacteria and algae (Chau et al., 2011, Vlamis et al., 2015, Wu et al., 2005, Yasumoto et al., 1986), and conversely compelling experiments indicating TTX could be produce endogenously by some organisms (Cardall et al., 2004, Daly et al., 1997). Tetrodotoxin was predominantly found in tropical regions (Lange, 1990) but in recent years, it has been detected in a greater number of species from temperate regions including: bivalves from the Mediterranean Sea (Vlamis et al., 2015) and England (Turner et al., 2015); and bivalves, gastropods and platyhelminths in New Zealand (McNabb et al., 2010, Salvitti et al., 2015). Increasing reports of TTX in farmed aquaculture species, such as bivalves, has drawn attention to the toxin, reinvigorating scientific interest and regulatory concerns (Knutsen et al., 2017).

The first report of TTX in bivalves was in the early 1990s when it was detected in the digestive glands of the Japanese scallops (*Patinopecten yessoensis;* 8 μg kg^−1^; Kodama et al., 1993). In 2011, McNabb et al. (2014) recorded high concentrations (800 μg kg^−1^) in an endemic clam (*Paphies australis*) in New Zealand. The identification of high concentrations in edible shellfish triggered further research on TTX in bivalves globally. The neurotoxin has now been detected in mussels (*Mytilus edulis*) from the UK (Turner et al., 2015) and Greece (Vlamis et al., 2015); in oysters (*Crassostrea gigas*) from the UK (Turner et al., 2015, Turner et al., 2017b); and in oysters and clams (*Mercenaria mercenaria*) from the Netherlands (Knutsen et al., 2017, Turner et al., 2017b). In 2015, researchers in China detected trace concentrations in clams (*Ruditapes philippinarum*) purchased from markets (Zhang et al., 2015). There are multiple attributes of bivalves that make them an ideal organism to investigate the source and dynamics of TTX, including; they are stationary and occur in relatively confined shallow sub-tidal areas making obtaining high number of individuals plausible and allowing systematic sampling of benthic communities; they can be maintained, and in many instances reared in captivity allowing long-term or manipulative experiments; and as filter feeders they occur low in the trophic pyramid (2nd order).

The detection of TTX in *P. australis* in New Zealand has led to concerns about health risks for human consumers and prompted further research as they are a common non-commercial shellfish species consumed in the country. Biessy et al. (2018) showed that TTX is primarily located in the siphons of *P. australis*, although low concentrations were also detected in the foot, digestive system, gills and labial palps. McNabb et al. (2014) detected the neurotoxin in individuals from several locations around New Zealand, however, there is uncertainty as to whether all *P. australis* populations in New Zealand contain TTX, and how variable concentrations are within and between locations. In a study on the TTX-containing sea slug *Pleurobranchaea maculata*, Wood et al. (2012b) showed marked variability in toxin concentrations within populations, with a general pattern of decreasing toxin content between populations along a north to south latitudinal gradient. This led the authors to speculate that TTX may be sourced exogenously in this species, with ocean currents, such as those that flow through Cook Strait (the water body that separates the North and South Islands of New Zealand) acting as a barrier for the producer. Studies in the northern hemisphere also indicate a potential latitudinal gradient or link to warmer water temperatures, with higher TTX concentrations in bivalves during summer months (Turner et al., 2017b) and in places with warmer waters (Vlamis et al., 2015).

A further important knowledge gap, which would enhance TTX management and risk assessment, is information on depuration rates of the neurotoxin in harvested shellfish. This data would assist in providing guidance on when it may be safe to recommence shellfish harvesting if a contamination event occurred. To our knowledge, no studies have investigated TTX depuration in shellfish or bivalves in a controlled environment. However, research on other marine biotoxins has shown that depuration rates vary among toxins and bivalve species, and range from days to several months (Lee et al., 2008). Depuration of TTX has been investigated in *Pl. maculata*, and a significant decrease in concentrations was observed over 126 days with the toxin migrating to the gonads, and largely depurating through laying of egg masses (Wood et al., 2012a).

The overarching aims of the present study were: (1) to investigate TTX concentrations within and between *P. australis* populations around New Zealand; (2) to determine whether depuration of TTX occurs in *P. australis* kept in captivity for 150 days and fed a TTX-free diet, and (3) to explore if TTX migrates between *P. australis* organs over a 150-day period. We hypothesised: (1) that a north to south latitudinal gradient of decreasing TTX exists, as observed in *Pl. maculata* (Wood et al., 2012b), and (2) that in *P. australis,* the neurotoxin migrates over time from organs involved in filtering or digestion to the siphon, where it functions as a chemical defence mechanism. Increasing knowledge on the distribution and within organism transport of TTX in the sessile *P. australis* may provide new insights to the toxin's origin and function in this species.

## 2 Materials and methods

### 2.1 Distribution of TTX in *Paphies australis* around New Zealand

**Fig. 1:**
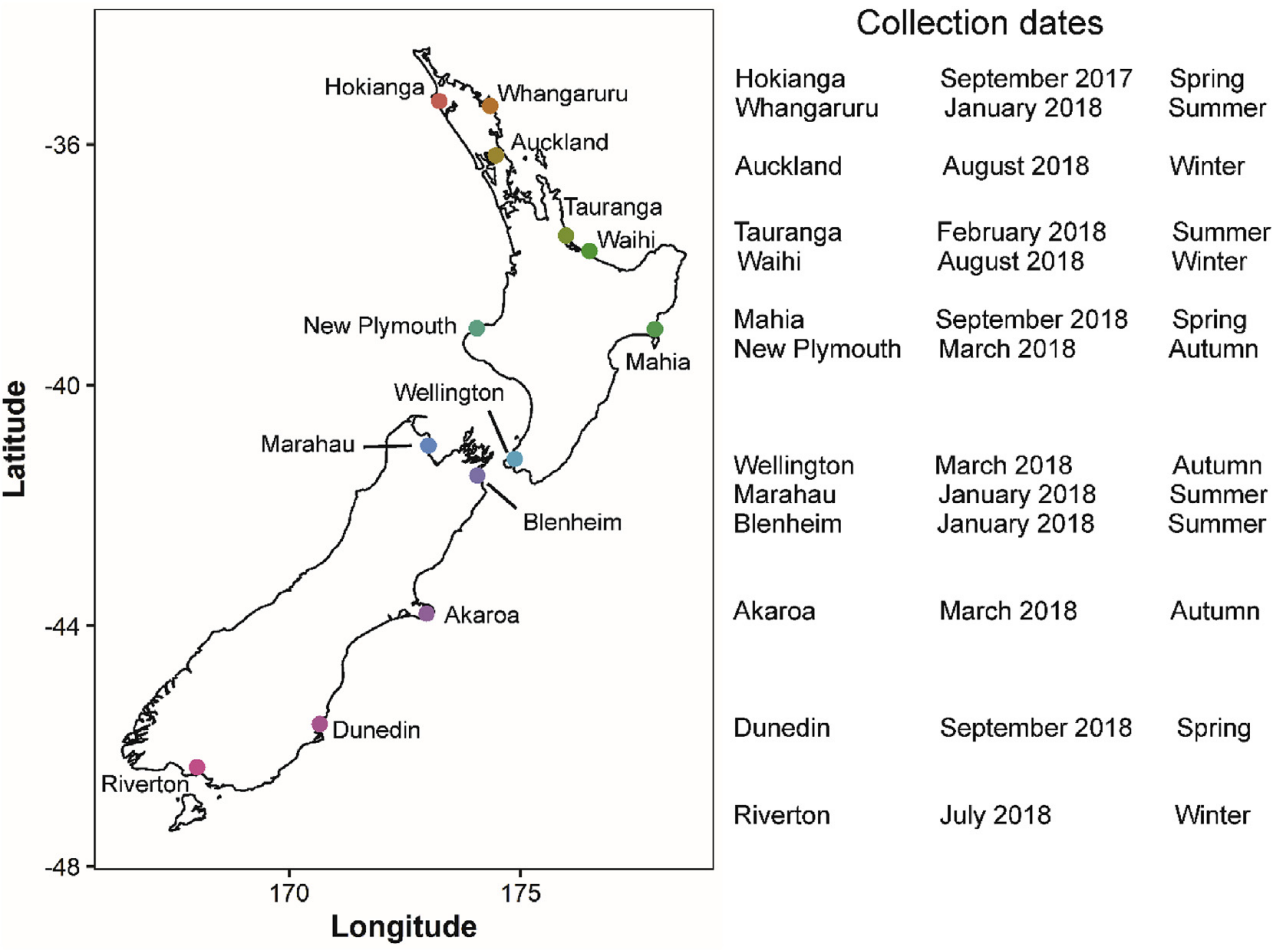
*Paphies australis* collection locations, dates and seasons from around New Zealand.

*Paphies australis* (n = 5 or 15) were collected from thirteen sites around New Zealand between September 2017 and September 2018 (Fig. 1; Supplementary information Table 1). The *P. australis* were rinsed in seawater, chilled (ca. 8 °C) and sent overnight to the laboratory (Cawthron Institute, Nelson, New Zealand). Once in the laboratory, five individuals were rinsed with Milli-Q water, and stored frozen (−20 °C) until later TTX analysis. Toxin extractions were not possible on individual organs because of their small sizes (a minimum of 300 mg of material is required). When possible, ten of the fifteen individuals were aseptically dissected, and the tissues pooled into four groups: the pair of siphons, foot, digestive gland, and the ‘rest’ which mostly included the mantle, gills, adductor muscles and gonads. The pooled samples were frozen (−20 °C) for later TTX analysis.

### 2.2 Depuration study

#### 2.2.1 *Paphies australis* collection and maintenance

*Paphies australis* (n = 435) were collected from the Hokianga Harbour (Northland, New Zealand, 35°28′S, 173°24′E; Fig. 1) on 28 September 2017 and placed in a metal shellfish collection basket. *Paphies australis* from this location had previously been shown to contain TTX (Biessy et al., 2018). Individuals were rinsed in seawater and placed inside an insulated container (9–12 °C) for 24 h while being transported to the laboratory. Once in the laboratory, *P. australis* were rinsed with sterile seawater and 15 individuals were dried and stored frozen (−20 °C) until later TTX analysis. The remainder were kept in two separate aerated aquariums (60 L), each containing 210 individuals. The aquariums were maintained at 18 ± 1 °C with a 14:10 h light:dark cycle and the water was recirculated through the two aquariums. The bivalves were fed *Isochrysis galbana* (2 L; ca. 12 × 10^6^ cells mL^−1^) every second day for 150 days. The aquariums were cleaned, and the water changed weekly to maintain the dissolved oxygen levels (7–8 mg/L) and salinity (34–35‰).

#### 2.2.2 Tetrodotoxin depuration study

Individuals (n = 15) were harvested from the tanks every third day for 15 days, every sixth day for 57 days, and then every twelfth day until the conclusion of experiment at day 150. After collection, *P. australis* were rinsed with Milli-Q water and 10 of the 15 individuals were aseptically dissected and the tissues pooled into four groups as above: the pair of siphons, foot, digestive tract and the ‘rest’. The pooled samples and the remaining five ‘whole’ *P. australis* were frozen (−20 °C) for later TTX analysis. Subsamples of *I. galbana* prior to feeding (50 mL) and faeces from the bottom of the aquariums were collected weekly. These samples were centrifuged (3000×*g*, 5 min), the seawater decanted and the pellet frozen (−20 °C) for later analysis. Negative extraction controls (Milli-Q water) were also extracted, following the extraction protocol described below, and analysed for TTX.

#### 2.2.3 Tetrodotoxin extraction and analysis

The following protocol was adapted from Biessy et al. (2018). Each sample (whole organism or organs of *P. australis*, algae, faeces and controls) was weighed (ca. 0.3–3.0 g), cut into small pieces (*P. australis* only) with a sterile blade and placed in a sterile tube (50 mL) with a corresponding volume (ca. 300–3000 μL) of Milli-Q water containing 1% acetic acid. Samples were homogenized (Ultra-Turrax^®^, IKA^®^, NC, USA) for 45 s to ensure complete homogenization. The tubes were boiled (5 min) and cooled in an ice bath (5 min) before briefly vortexing. Samples were centrifuged (3200×*g*, 10 min) and 0.5–1 mL of the supernatant transferred to a centrifuge tube (1.7 mL) containing 25% ammonia (2.5–5 μL; Honeywell, Seelze, Germany). Samples were then centrifuged (17,000×*g*, 1 min) and the supernatant subjected to the graphitised carbon solid phase extraction (SPE) method as described in Boundy et al. (2015) using Supelclean ENVI-Carb 250 mg/3 mL cartridges (Sigma-Aldrich, MO, USA). Tetrodotoxin was analysed and quantified by liquid chromatography tandem-mass spectrometry analysis as described by Turner et al. (2017a).

#### 2.2.4 Statistical analysis

Statistical analyses were performed using the R statistical package (R Development Core Team, 2014). Normality was checked through inspection of Quantile-Quantile plots and conducting a Shapiro-Wilk test. Normality was improved by log transformation of the spatial data. Statistical differences in TTX concentrations between sites was assessed using a one-way analysis of variance (ANOVA). A Tukey's honestly significant difference (HSD) post-hoc test was used to identify which sites were responsible for the significant differences. Levene's test was used to assess the equality of variance in TTX concentrations between sites (Carroll and Schneider, 1985).

A linear regression model was constructed to investigate the relationship between log transformed total TTX concentrations in *P. australis* and time (day) of the experiment. Generalized additive models (GAMs; Hastie and Tibshirani, 1990) were used to model nonlinear trends in organ TTX concentrations in relation to time (days). Models were selected with a stepwise procedure based on the general Akaike information criterion (AIC) and were validated by inspecting the deviance residuals. The GAMs models were run with the *mgcv* package in R (Wood and Wood, 2015).

## 3 Results

### 3.1 Distribution of tetrodotoxin in *Paphies australis* around New Zealand

**Fig. 2:**
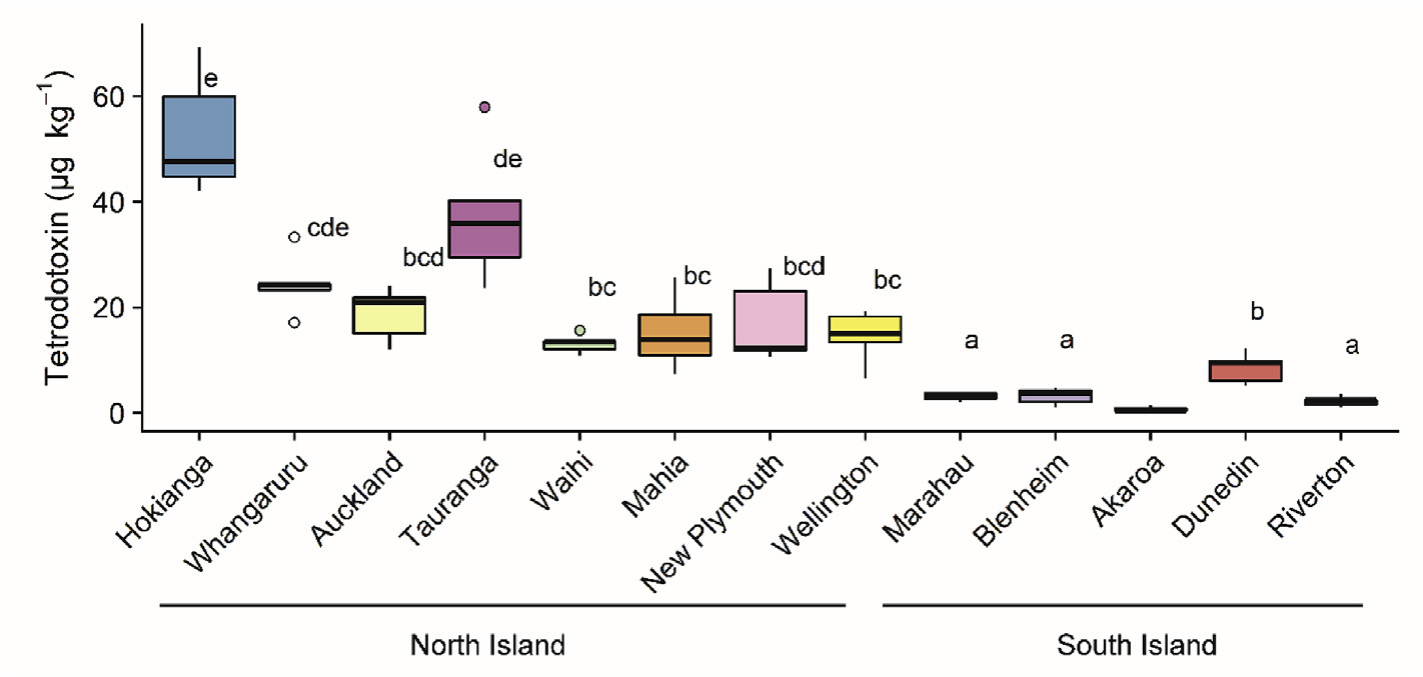
Tetrodotoxin concentrations in populations of *Paphies australis* collected around the New Zealand coastline, determined using liquid chromatography-mass spectrometry (n = 5). Solid black line shows median, box shows 1st and 3rd quartiles, whiskers extend to the last data point within 1.5 times the inter-quartile range. Dots outside the whiskers are considered as outliers. Different letters indicate where significant differences occur between sites (one-way ANOVA with Tukey's HSD post-hoc test, *p* < 0.001). Sites are ordered by increasing latitude for each Island (i.e., North and South Island). Akaroa was not included in the statistical as the concentrations of toxin were under the limit of quantification of the method (<2.0 μg kg^−1^).

Tetrodotoxin was detected in *P. australis* from all sites around New Zealand (Fig. 2). The samples from Akaroa were under the limit of quantification of the method (<2.0 μg kg^−1^) but the chromatograms clearly indicated trace levels of TTX were present in these samples. Tetrodotoxin was the main congener (>99%) detected in all samples analysed.

One-way ANOVA showed a significant difference between TTX concentrations among all sites (*F* = 39.2, *p* < 0.001), with a Tukey's HSD post-hoc test identifying a complex overlap between sites (Fig. 2 and Supplementary information Table 2). The highest concentrations were measured in *P. australis* from the northernmost site, the Hokianga Harbour (median concentration 47.6 μg kg^−1^). Median toxin concentrations in *Paphies australis* from the South Island sites were significantly lower (Marahau, 3.2 μg kg^−1^; Blenheim, 3.8 μg kg^−1^; Dunedin, 8.5 μg kg^−1^; Riverton, 2.4 μg kg^−1^) than the North Island sites (Hokianga Harbour; Whangaruru Harbour, 24.3 μg kg^−1^; Auckland, 20.9 μg kg^−1^; Tauranga, 35.9 μg kg^−1^; Waihi 13.5 μg kg^−1^; Mahia, 17.3 μg kg^−1^; New Plymouth, 12.3 μg kg^−1^; and Wellington, 15.0 μg kg^−1^). A Levene's test showed that the degree of variance did not differ among sites (*F* = 0.62).

**Fig. 3:**
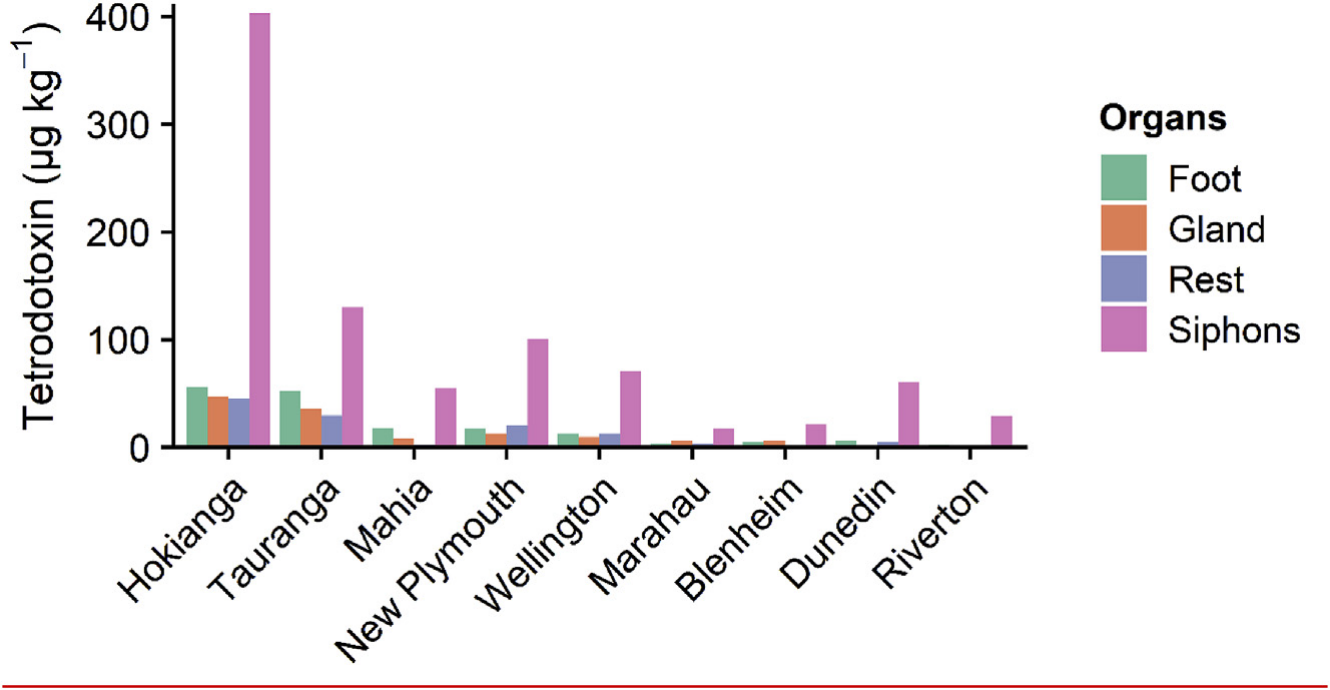
Tetrodotoxin concentrations in the organs and tissues of *Paphies australis* from different sites around New Zealand. Data are a composite of organs from 10 different individuals pooled together to enable sufficient mass for the toxin extraction. Organ dissections and extractions were not possible from sites where less than 15 *P. australis* were collected (i.e., Whangaruru, Auckland, Waihi and Akaroa sites). Sites are ordered by increasing latitude for each island (i.e., North and South Islands).

At all sites, and regardless of TTX concentrations in whole *P. australis*, the siphons contained the highest amount of toxin compared to the other tissue types (Fig. 3). *Paphies australis* from the North Island populations also contained TTX in their foot, digestive gland and mantle (except in the Mahia samples). Tetrodotoxin was detected in low concentrations (ca. 6 μg kg^−1^) in the digestive glands and of *P. australis* from two of the South Island sites (Marahau and Blenheim) and in the mantle from the Dunedin samples (Fig. 3).

### 3.2 Tetrodotoxin depuration

#### 3.2.1 Depuration of tetrodotoxin in *Paphies australis* maintained in captivity

*Paphies australis* stayed healthy for the duration of the experiment, except for six individuals that died and were removed from the aquariums. The feed was consumed within 4 h when placed in the aquariums, showing that the bivalves were healthy and feeding well. All negative extraction controls (Milli-Q water), *I. galbana* and faeces samples were negative for TTX. Tetrodotoxin was the main congener (>99%) detected in all samples analysed over the entire study.

**Fig. 4:**
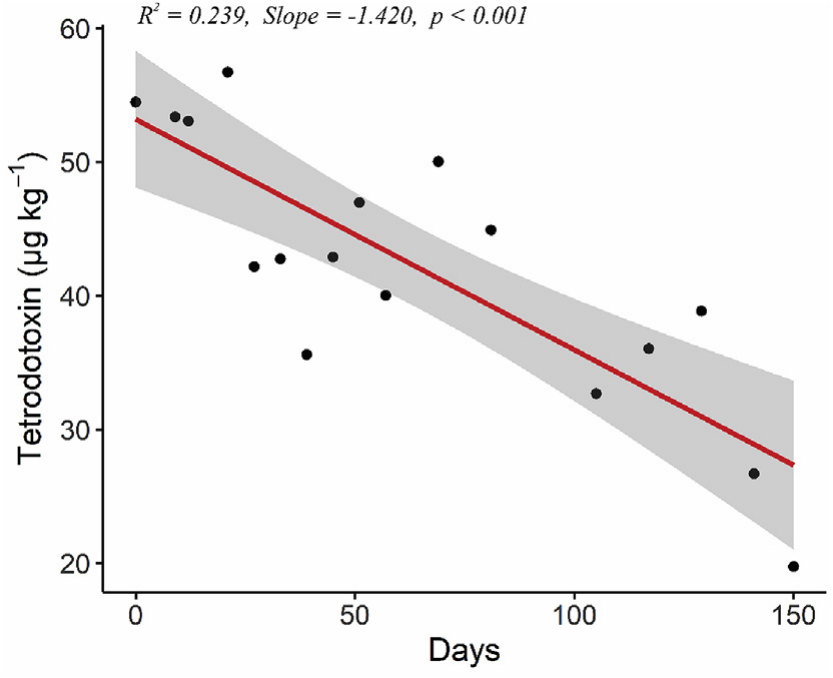
Tetrodotoxin (TTX) concentrations in *Paphies australis* maintained in captivity and fed a TTX-free diet for 150 days. Data are averages from five individuals. The linear model indicated a significant (*p* < 0.001) decrease in TTX concentrations after 150 days.

The average TTX concentration in *P. australis* at day 0 was 56.5 ± 5.7 μg kg^−1^ and declined to 21.7 ± 6.3 μg kg^−1^ after 150 days in captivity (Fig. 4). A linear model was fitted to the data (*R*^*2*^ = 0.24, *F* = 28.05, *p* < 0.001) which showed an average depuration rate of TTX over the entire experiment in *P. australis* of 0.23 μg kg^−1^ per day.

#### 3.2.2 Changes in TTX concentrations in the organs of *Paphies australis*

**Fig. 5:**
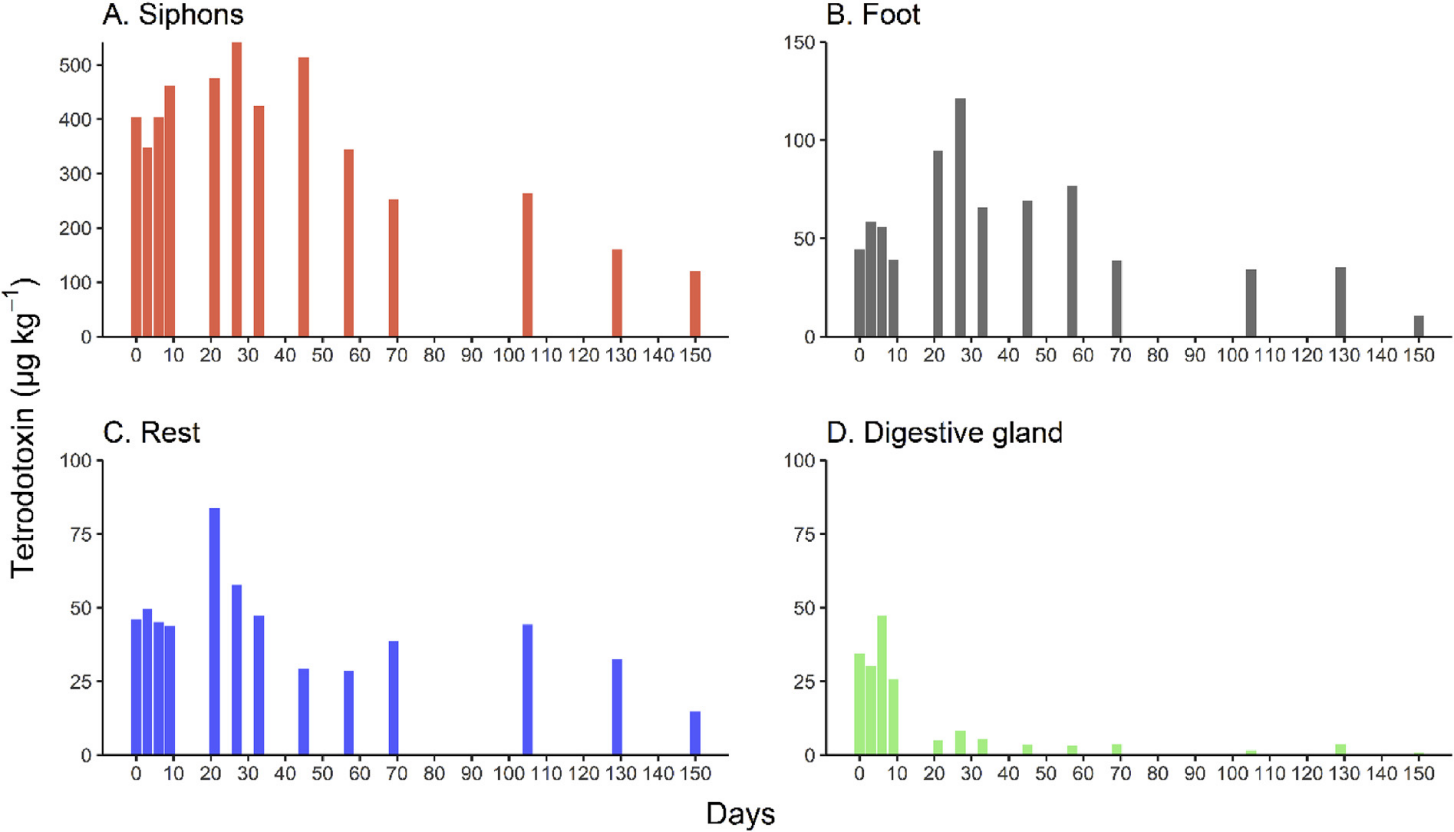
Tetrodotoxin (TTX) concentrations, in the organs of *Paphies australis* maintained in captivity and fed a TTX-free diet for 150 days, measured using liquid chromatography-tandem mass spectrometry. Results at each time point are a total of 10 pooled *P. australis* individuals (n = 1).

Overall the depuration pattern was relatively consistent across all organs with a decline in TTX concentrations over 150 days. At day 0, the siphons contained the highest concentrations of TTX (404 μg kg^−1^, Fig. 5). Low concentrations (35–45 μg kg^−1^) were also detected in the foot, digestive gland and mantle. In general, the TTX concentrations increased from day 0 until day 28 in the siphons, foot and mantle. Concentrations in these organs then remained relative stable until day 45 for the siphons and in the foot until day 28 after which concentrations decreased (Fig. 5A and B). The TTX concentrations remained relatively constant in mantle, with notably lower concentrations recorded on the final day of the experiment (150; Fig. 5C). In contrast, after 10 days, the digestive gland contained only traces of TTX (<5.1 μg kg^−1^; Fig. 5D). The GAMs showed that there was a significant (*p* < 0.001) decrease in TTX concentrations in all the organs over the 150-day experiment. Tukey HSD pairwise comparisons demonstrated that the average TTX concentrations over the experiment was significantly different between all four organs (*p* < 0.001), except between the mantle and foot (*p* = 0.76).

## 4 Discussion

### 4.1 TTX concentrations in *Paphies australis* populations around New Zealand

All *P. australis* populations sampled around New Zealand contained TTX, albeit only trace amounts were detected in the Akaroa samples. However, there was marked variability in toxin concentrations between regions, with sites from the North Island containing significantly higher amounts than those from the South Island. Wood et al. (2012b) found a similar north-south pattern during their study on TTX in the sea slug *Pl. maculata*. Two plausible explanations for the north-south gradient in toxin concentrations are the difference in the abundance of the TTX source, should it be exogenous; or genetically disconnected populations, should the source be endogenous.

The Cook Strait is a 70-km wide body of water separating the North and South Islands of New Zealand (Bowman et al., 1983). The winds are highly variable, often leading to sharp accelerations and gale conditions (Brodie, 1960), resulting in Cook Strait having some of the world's strongest tidal and ocean currents, which may limit the exchange of planktonic organisms resulting in different diets between North and South Island populations (Walters et al., 2010).

The strong currents of Cook Strait also significantly alter the structure of the sea shelf which can have significant biological consequences (Bowman et al., 1983). For example, natural populations of bivalves such as *Paphies subtriangulata* and *Perna canaliculus* from the North and South Islands show distinct genetic differentiations (Gardner et al., 2010, Ross et al., 2009). Studies on the population genetics of *P. australis* suggest three distinct genetic groups (Northern, South Eastern and South Western groups; Hannan, 2014). Thus, a further possible reason for varying TTX-concentrations among geographic regions is that the genetically different groups may have varying propensities to uptake and accumulate TTX.

The trend for increasing concentrations of TTX with decreasing latitudes (i.e., moving closer to the equator) and warmer water has been observed previously with higher TTX concentrations recorded in the sunniest and warmest parts of southern England (Turner et al., 2017b). In Europe, high TTX has also been found in warm waters, including Greece (Vlamis et al., 2015) and Portugal (Rodriguez et al., 2008, Silva et al., 2012). The latitudinal/temperature associated patterns identified in the present study, in concert with the relationship with warmer climates in the Northern Hemisphere, could indicate the presence of a warm-water-adapted TTX-producer, or that the biosynthesis of TTX or the synergistic relationships between the TTX-producer and host organism is enhanced under these conditions (Pratheepa et al., 2016). In this study, the Waihi estuary and the Tauranga sampling sites had significantly (*p* = 0.008) different concentrations of TTX despite being less than 100 km apart. In this study, samples were collected at different times of the year: Tauranga in summer (February) and Waihi in winter (August). These results correlate with the hypothesis that a TTX producer might be most prolific in warmer waters, or that TTX synthesis is upregulated at hotter temperatures.

As global sea temperature rises, there may be a pressing need to enhance monitoring of TTX in edible marine species in locations where the toxin has traditionally been absent. The European Food Safety Authority recently released a scientific opinion (Knutsen et al., 2017) on the risk to public health related to the presence of TTX in marine bivalves and gastropods. They suggest that TTX levels above 44 μg kg^−1^ in bivalves would be a concern for consumers when a large portion size (>400 g) is consumed. In the present study, the *P. australis* from the Hokianga Harbour (median concentration 47.6 μg kg^−1^) are above this threshold but there have not been any official reports of intoxication from this site to date.

Statistical analyses of the organ data were not possible because the organisms collected were usually small (<25 mm long) and once dissected there was not enough biomass for individual analysis, thus the five samples were pooled. However, the results from all sites consistently demonstrated that the siphons contained the highest amounts of TTX. These results concur with the study from Biessy et al. (2018), where immunohistochemistry was used to show the presence of TTX within cells in the siphons, and LC-MS/MS to demonstrate that toxin concentrations (on a per mass basis) were significantly higher in the siphon than other organs. Other clam species like *Paphies subtriangulata* and *Saxidomus gigantea* have been shown to sequester saxitoxin, another neurotoxin with similar mode of action and toxicity to TTX, in their siphons (MacKenzie et al., 1996, Smolowitz and Doucette, 1995). As noted by Biessy et al. (2018), the accumulation of TTX in siphon tissue could suggest that TTX is present in the seawater or within planktonic organisms that are filtered while feeding, possibly indicating that the toxin comes from an exogenous source.

### 4.2 TTX concentrations in *P. australis* maintained in captivity

This study demonstrated that when *P. australis* containing TTX are kept in a controlled environment and fed a TTX-free diet, the toxin significantly depurated over 150 days. Bivalve species can be classified as rapid (weeks to detoxify; up to 15% toxin loss day^−1^) or slow detoxifiers (months to years to detoxify; > 3% loss day^−1^). When averaged out of over the 5 months of the experiment, the depuration rate of TTX in *P. australis* was 0.23 μg kg^−1^ per day (0.41% loss day^−1^), classifying them as a slow detoxifier for TTX (Bricelj and Shumway, 1998). Toxin biotransformation, which may lead to changes in net toxicity, varies greatly among species and between biotoxins. Some species exhibit rapid enzymatic decarbamoylation (e.g., the clam *Protothaca staminea* in presence of saxitoxin), whereas other bivalves (e.g., *M*. *edulis*) show limited toxin metabolism and thus are useful indicators of the toxigenic source (Bricelj et al., 2012).

Similar studies on TTX persistence and depuration in marine and terrestrial organisms fed non-toxic diets concur with the findings of the present study. Wood et al. (2012a) demonstrated that the TTX concentrations in the sea-slug *Pl. maculata* significantly decreased overtime when kept in aquariums for 126 days with the stomach depurating the fastest (6.7 μg kg^−1^ day^−1^). Yotsu-Yamashita et al. (2012) showed that newts (*Notophthalmus viridescens*) lost their toxin after being kept in captivity and fed a TTX-free diet over several years. Recently, Turner et al. (2017b) investigated the depuration of TTX in wild populations of marine bivalves in the field and found that mussels (*M. edulis*) and oysters (*C. gigas*) rapidly depurated the toxin with 75% of the total lost in four weeks (from 80 to 20 μg kg^−1^), though this in uncontrolled conditions. The authors also showed that uptake and depuration patterns were species-specific. One important consideration with the Turner et al. (2017b) study is that it was a non-controlled field study and the difference in TTX concentrations might be due to the natural variability between individuals, and the source of TTX may still be present in the environment and being accumulated, making accurately measuring depuration rates difficult. In a recent study, Biessy et al. (2018) demonstrated that TTX was mostly stored in the siphons of *P. australis*, potentially a defensive strategy (Kvitek, 1991), but it is important to note that not all bivalves have functioning siphons. Those that live on or above the substrate (e.g., *M. edulis* or *C. gigas*) do not need protruding siphons (Gibson et al., 2008), which could explain why some species might hold onto TTX more than others.

Tetrodotoxin was detected in all organs of *P. australis* investigated in the present study, and in the test animals the siphons again contained the highest amount of toxins, which remained the case for the entire study period. As noted above, because of the small size of the organs and the need to pool them to obtain a sufficient sample for TTX analysis. This also makes it challenging to determine if patterns such as the increase in TTX concentrations in the digestive glands and the mantle (only one point in time) were a real shift, analytical or natural variability. The increase in TTX concentrations in the siphons and the foot was more gradual (over six time points) and suggests a migration of the toxin between organs, possibly from the digestive tract and mantle to the siphons and/or the foot. Only one other study has investigated TTX depuration from different organs: Wood et al. (2012a) showed that the slugs *Pl. maculata* contained TTX in all their organs with the highest concentrations initially occurring in the stomach and migrating to the gonads, which is then transferred to the eggs, where TTX may act as a chemical defence deterring predators from eating the egg masses. Other studies on marine organisms have also proposed that the ecological function of TTX is a chemical defence (reviewed in Williams, 2010). For instance, the snails *Natica lineata* released TTX from their ‘muscle cavity’ upon disturbance (Hwang et al., 1990); some species of pufferfish secrete TTX in their skin after electrical stimulation (Kodama et al., 1985); and gonads (i.e., ovaries, oocytes, eggs) of many toxic puffer fishes and larvae of flatworms often harbour the highest concentrations of TTX (Williams, 2010).

Tetrodotoxin has previously been detected in the intestinal epithelium, the rectum and the stomach wall of *P. australis* (Biessy et al., 2018), leading us to hypothesise that the bivalves might obtain TTX from their diet and that over a few days or weeks, the toxin might migrate to other organs such as the foot and siphons. Over the experimental period, TTX did also depurate from the foot and siphons, potentially indicating that *P. australis* are not endogenously producing TTX, rather they accumulate the toxin from an exogenous source. Previous studies have shown that TTX can accumulate through the food chain: for example, Lin and Hwang (2001) demonstrated the starfish *Astropecten scoparius* accumulates TTX from consuming the TTX-containing gastropod *Umbonium suturale*. Kono et al. (2008) demonstrated that the non-toxic pufferfish *Fugu niphobles* accumulated TTX when fed a highly toxic diet and recent study (Itoi et al., 2018) confirmed that the pufferfish *Takifugu niphobles* becomes toxic after feeding on toxic flatworms. The mechanism of depuration in marine bivalves remains unknown but it is possible that the molecule is broken down and/or metabolised inside the organisms at a slow rate.

We cannot completely rule out the possibility that *P. australis* endogenously produces TTX and that this is enhanced when exposed to certain abiotic or biotic triggers (e.g., pH, temperature, predators), a pattern which has been shown for toxin production in other bacteria and microalgae. For instance, hepatotoxin production in cyanobacteria is thought to be influenced by parameters such as pH, light or temperature (Neilan et al., 2013); and brevetoxin production can be triggered by a decrease in salinity in the dinoflagellate *Karenia brevis* (Errera and Campbell, 2011). However, the results from the spatial and the depuration studies undertaken to date provide compelling evidence to support the hypothesis of an exogenous source of TTX in *P. australis*. When collated with results from similar depuration studies in other marine organisms such as sea slugs (Wood et al., 2012a) or puffer fish (Noguchi et al., 2006), which span a range of trophic levels, live in varying habitats, in distinct parts of the globe, and whose diets would be markedly different from *P. australis,* the evidence suggests an ubiquitous microbial source as the likely producer; a suggestion that has been touted for many decades but not conclusively proven (Bane et al., 2014, Khor et al., 2013, Magarlamov et al., 2017). Because *P. australis* are stationary filter feeders, they may prove a more amenable organism to study TTX-containing dietary organisms. Although the source of TTX may be relatively prevalent, we suggest not all organisms can accumulate this toxin, for example, when other New Zealand clam species including *Austrovenus stutchburyi* and *Paphies subtriangulata*, were collected at the same sites as toxic *P. australis* individuals as part of this study, no TTX was detected (Biessy, unpublished data). We hypothesise that *P. australis* contains unique TTX-binding proteins similar to those found in the puffer fish *Fugu pardalis* (Yotsu-Yamashita et al., 2001) or the crab *Hemigrapsus sanguineus* (Nagashima et al., 2002), that allow them to store the toxin in different organs.

## Conflict of interest

The authors declare no conflict of interest.

## Funding

This work was supported by; the MBIE-funded Safe New Zealand Seafood Research Programme (contract No.: CAWX1801), a PhD scholarship from the New Zealand Food Safety Science & Research Centre to Laura Biessy, and the Cawthron Institute Internal Investment Fund.
