# Supplementary material for: Spatial variability and depuration of tetrodotoxin in the bivalve Paphies australis from New Zealand
Source: Toxicon X. 2019 Feb 23;2:100008. doi: 10.1016/j.toxcx.2019.100008 (PMC7286059; doi:10.1016/j.toxcx.2019.100008)
Supplement: Multimedia component 1 [file mmc1.docx]

**Supplementary Material**

**Table 1.** Dates and coordinates of *Paphies australis* collection sites from around New Zealand.

| **Sites** | **Region** | **Collection Date** | **Coordinates** | |
| --- | --- | --- | --- | --- |
|  |  |  | **Latitude** | **Longitude** |
| **Hokianga Harbour** | Northland (West Coast) | 28 September 2017 | 35°47’28” S | 173°40’23” E |
| **Whangaruru Harbour** | Northland (East Coast) | 20 January 2018 | 35°36’18” S | 174°34’03” E |
| **Auckland (Whangateau Harbour)** | Auckland | 26 August 2018 | 36°18’35” S | 174°46’44” E |
| **Tauranga (Kauri Point)** | Bay of Plenty/Waikato | 13 February 2018 | 37°51’56” S | 175°97’78” E |
| **Waihi Estuary** | Bay of Plenty | 28 August 2018 | 37°76’41” S | 176°48’32” E |
| **Mahia (Oraka)** | Hawkes Bay | 24 September 2018 | 39°07’23” S | 177°89’96” E |
| **Port Taranaki** | New Plymouth | 28 March 2018 | 39°05’82” S | 174°05’01” E |
| **Petone Beach** | Wellington | 22 March 2018 | 41°13’39” S | 174°52’15” E |
| **Marahau** | Nelson Tasman | 25 January 2018 | 41°00’88” S | 173°00’96” E |
| **Bleinhem (Wairau River)** | Marlborough | 24 January 2018 | 41°50’36” S | 174°05’94” E |
| **Akaroa (Children’s Bay)** | Canterbury | 18 March 2018 | 43°80’08” S | 172°96’57” E |
| **Dunedin (Karitane)** | Otago | 18 September 2018 | 45°63’48” S | 170°65’51” E |
| **Riverton** | Southland | 29 July 2018 | 46°21’38” S | 168°12’98” E |
|  |  |  |  |  |

**Table 2.** Pairwise *post hoc* comparisons (Tukey HSD test) of mean tetrodotoxin concentrations among New Zealand populations of *Paphies australis*. Bolded values represent statistically significant differences (*p* < 0.05).

| **Sites** | Whangaruru | Auckland | Tauranga | Waihi | Mahia | New Plymouth | Wellington | Marahau | Blenheim | Dunedin | Riverton |
| --- | --- | --- | --- | --- | --- | --- | --- | --- | --- | --- | --- |
| Hokianga | 0.098 | **0.004** | 0.940 | **<0.001** | **0.001** | **<0.001** | **<0.001** | **<0.001** | **<0.001** | **<0.001** | **<0.001** |
| Whangaruru |  | 0.995 | 0.899 | 0.389 | 0.946 | 0.881 | 0.490 | **<0.001** | **<0.001** | **<0.001** | **<0.001** |
| Auckland |  |  | 0.234 | 0.973 | 0.999 | 0.999 | 0.989 | **<0.001** | **<0.001** | **0.008** | **<0.001** |
| Tauranga |  |  |  | **0.007** | 0.103 | 0.064 | **0.011** | **<0.001** | **<0.001** | **<0.001** | **<0.001** |
| Waihi |  |  |  |  | 0.998 | 0.999 | 1 | **<0.001** | **<0.001** | 0.266 | **<0.001** |
| Mahia |  |  |  |  |  | 1 | 0.999 | **<0.001** | **<0.001** | **0.025** | **<0.001** |
| New Plymouth |  |  |  |  |  |  | 0.999 | **<0.001** | **<0.001** | **0.042** | **<0.001** |
| Wellington |  |  |  |  |  |  |  | **<0.001** | **<0.001** | 0.195 | **<0.001** |
| Marahau |  |  |  |  |  |  |  |  | 1 | 0.078 | 0.894 |
| Blenheim |  |  |  |  |  |  |  |  |  | **0.035** | 0.975 |
| Dunedin |  |  |  |  |  |  |  |  |  |  | **<0.001** |
